# A Novel Donkey Milk–derived Human Milk Fortifier in Feeding Preterm Infants: A Randomized Controlled Trial

**DOI:** 10.1097/MPG.0000000000002168

**Published:** 2018-10-24

**Authors:** Enrico Bertino, Laura Cavallarin, Francesco Cresi, Paola Tonetto, Chiara Peila, Giulia Ansaldi, Melissa Raia, Alessia Varalda, Marzia Giribaldi, Amedeo Conti, Sara Antoniazzi, Guido E. Moro, Elena Spada, Silvano Milani, Alessandra Coscia

**Affiliations:** ∗ Neonatal Unit, University of Turin, City of Health and Science of Turin, Turin; † Institute of Sciences of Food Production, Italian National Research Council, Grugliasco; ‡ Research Centre for Engineering and Agro-Food Processing, Council for Agricultural Research and Economics (CREA), Turin; § Italian Association of Human Milk Banks; || Laboratory of Medical Statistics, Biometry and Epidemiology “GA Maccacaro”, Department of Clinical Sciences and Community Health, University of Milan, Milan, Italy.

**Keywords:** bovine milk, donkey milk, feeding intolerance, human milk fortifier, very low birth weight infants

## Abstract

**Objectives:**

The purpose of the present randomized controlled clinical trial was to compare the use of donkey milk–derived fortifier (DF) with commercial bovine milk–derived fortifier (BF) in very preterm or very-low-birth-weight newborns, in terms of feeding tolerance.

**Methods:**

This trial included 156 newborns born at <32 weeks of gestational age and/or with a birth weight ≤1500 g. Newborns were randomized 1:1 to receive enteral feeding with either a BF-arm, or a new, DF-arm for 21 days. The fortification protocol was the same for both study arms, and the 2 diets were designed to be isoproteic and isocaloric. Feeding tolerance was assessed by a standardized protocol.

**Results:**

The risk of feeding intolerance tended to be lower in DF-arm than in BF-arm, with a relative risk reduction of 0.63 (95% confidence interval: −0.29, +0.90). The mean number of episodes per newborn of feeding intolerance and feeding interruptions (any duration) were consistently lower in the DF-arm than in the BF-arm. Episodes of bilious gastric residuals and vomiting were significantly lower in the DF-arm. Time needed to reach full enteral feeding (150 mL · kg^−1^ · day^−1^) and daily weight increase between the first day of exclusive enteral feeding (ie, without administering intravenous fluids) and discharge were similar in the BF- and DF-arms.

**Conclusions:**

These results suggest that DF improve feeding tolerance when compared with standard bovine-derived fortifiers, with a similar auxological outcome.

**What Is Known**

- Human milk is the recommended food for preterm newborns; however, it has to be fortified.
- At present, the most common fortifiers are bovine milk derived. The optimal composition of human milk fortifiers is still debated.

**What Is New**

- A new donkey milk–derived human milk fortifier is suitable for feeding preterm and very-low-birth-weight newborns.
- The donkey milk–derived fortifier, compared to a bovine counterpart in an isocaloric and isoproteic diet, seems to improve feeding tolerance, with a similar auxological outcome.

Very preterm (gestational age <32 weeks) and very-low-birth-weight (VLBW, ie, <1500 g) newborns currently represent the majority of patients admitted to neonatal intensive care units (NICU) (1). Improvements in perinatal care have led to an increased survival rate in these newborns, which has offered new insights into their outcome and their health status in adulthood.

Nutrition is fundamental to neonatal survival and short-term outcomes, but it also has long-term consequences on quality of life in very preterm and VLBW newborns. Indeed, these newborns require adequate qualitative and quantitative nutrition, particularly in terms of protein intake, the lack of which is the main cause of postnatal growth deficits (2). Human milk is the recommended food for all neonates (3,4), but as breast milk alone does not meet the nutritional requirements of preterm newborns (5,6), it is supplemented with additional nutrients (7,8).

Fortification of human milk still represents a significant challenge (9–11), as concerns have been raised regarding fortification strategies and the composition of fortifiers. Individualized fortification is the current recommended strategy (12,13), and fortifiers must be composed of simple, high-quality, well-tolerated nutritional supplements. Recently, human milk–based fortifiers have been proposed, but their utilization is limited by high costs and ethical issues. Moreover, there is no strong evidence that human milk–based fortifiers in otherwise exclusively human milk–fed preterm infants affect important outcomes. (14).

Based on its physiochemical properties, milk from monogastric animals has been suggested to be more suitable than bovine milk for human nutrition (15). Donkey milk showed biological effects comparable with those elicited by human milk (16,17). Our hypothesis is that feeding very preterm and VLBW newborns with human milk supplemented with donkey milk–derived fortifiers (DFs) will improve feeding tolerance. Thus, the present trial compared the use of DF and commercial bovine milk–derived fortifier (BF) in very preterm and VLBW newborns, in terms of feeding tolerance and short-term auxological outcomes.

## METHODS

This study was performed in the NICU of Turin University. It was approved by Ethics Committee (AN: 0025847, 27/05/2014) and registered (*http://www.isrctn.com/ISRCTN70022881*, ISRCTN70022881) after the trial starting date. The study protocol was evaluated by JPGN Editorial Office. Recruitment period was 27/11/2014 to 22/12/2016. Written informed consent was obtained from the parents of all included newborns before enrollment.

### Study Population

The inclusion criteria were gestational age <32 weeks and/or birth weight ≤1500 g, exclusive feeding with human milk (own mother's milk or donor milk), and enteral feeding ≥80 mL · kg^−1^ · day^−1^ within the first 4 weeks of life. Newborns with severe gastrointestinal pathologies (necrotizing enterocolitis, colostomy, intestinal obstruction, symptoms of peritonitis, presence of blood in the feces), chromosomal abnormalities or major malformations, hereditary metabolic diseases, intravascular disseminated coagulopathy, shock, patent ductus arteriosus (PDA) requiring medical care or surgery at the time of randomization, and severe renal failure (serum creatinine >2 mg/dL) were excluded.

### Study Design

Eligible newborns were randomly allocated 1:1 into 2 arms in accordance with a list generated by a data step written in SAS (18) language: the BF-arm and the DF-arm. In the BF-arm, a bovine milk–derived commercial multicomponent fortifier (FM85, Nestlé) and a bovine milk–derived protein concentrate (Protifar, Nutricia, Utrecht, The Netherlands) were used. In the DF-arm a donkey milk–derived multicomponent fortifier and donkey milk–derived protein concentrate were used (FortiLat, Torino, Italy). The DF is not commercially available and was produced according to current EU legislation on food for special medical purposes.

All newborns received enteral feeding according to a regimen of *adjustable fortification*, based on blood urea nitrogen determination, for 21 days (19,20). The intervention started when the infants were able to tolerate a volume of ≥80 mL · kg^−1^ · day^−1^ (randomization time) and, according to study protocol, was planned to last 21 days; the intervention was suspended at discharge from the hospital for any reason (transfer, death, discharge home).

Please refer to our previous article (21) for a detailed description of the methodology used in the study. Because the protein concentration and energy content of bovine milk–derived products differ from those of donkey milk–derived products, the amounts of powder required to obtain the same level of fortification were different. Moreover, because the same nurses were in charge of both the preparation and administration of meals and the evaluation of feeding tolerance, this study must be regarded as an open-label trial. Increases in the quantity of milk given during enteral feeding were strictly regulated according to the feeding protocol adopted in the NICU, based on the evaluation of signs of feeding intolerance. Data on necrotizing enterocolitis that occurred after randomization, PDA, sepsis, mortality, hospital stay duration, intraventricular hemorrhage, and retinopathy of prematurity (defined according to the Vermont Oxford Network) (22) were collected from hospital records.

Babies were discharged from the hospital when they met all following criteria: satisfactory weight gain while receiving full oral feeding, maintenance of adequate thermal stability, and resolution of acute medical conditions.

### Outcome Measures of Feeding Tolerance

#### Primary Endpoint

Primary endpoint includes death, necrotizing enterocolitis, or at least 1 episode of feeding intolerance, defined as interruption of enteral feeding for at least 8 consecutive hours during the observation period.

#### Secondary Endpoints

Secondary endpoints include number of episodes of feeding intolerance, feeding interruption (any duration), bilious gastric residuals, vomiting, and total hours of enteral feeding interruption.

Time required to reach full enteral feeding (150 mL · kg^−1^ · day^−1^) and daily weight gain (weight-Δ standard deviation score (ΔSDS)/days) from the first day of exclusive enteral feeding (without administering intravenous fluids) until discharge were also evaluated.

### Study Size

The evaluation of the previous year's hospital records, carried out before the start of the present study, revealed that approximately 45% of very preterm or VLBW newborns admitted to the NICU had at least 1 episode of feeding intolerance (primary endpoint). A 25% reduction in the frequency of the primary endpoint was regarded as the minimum clinically important difference; under these assumptions, 62 newborns per arm had to be recruited to ensure an 80% study power, given a risk of type I error at the usual level of 5%. However, the occurrence of the primary endpoint was much lower than that assumed in the protocol, and no adverse effect of FortiLat was observed. Because the occurrence of primary endpoint in our study population resulted to be much lower than that assumed in the protocol, and no adverse effect was observed, when the planned study size was achieved, it was decided to continue the enrollment until the stock of FortiLat ran out. For this reason, we present information on the planned study with the initial 62 newborns per arm, and the extended study, with the additional recruitment. A further randomization list was generated for the extension of the study.

### Statistical Analysis

The analysis was performed on 2 populations:

- All randomized subjects (ARS) population, which included all randomized newborns.
- Per-protocol (PP) population, which included only newborns observed for 21 days in our hospital, and that actually received donkey milk or bovine fortifier according to the protocol, excluding, consequently, the babies transferred to other hospitals or discharged home before 21 days of observation.

The primary endpoints were evaluated both in the ARS and in the PP population. In the ARS population, the analysis (primary analysis) was performed in accordance with the intention-to-treat approach: failure included all the conditions that cannot be defined success, that is, occurrence of necrotizing enterocolitis, at least 1 episode of feeding intolerance, death, or transfer to another hospital before day 21 of observation. Subjects in the ARS population who were discharged home before the 21st day were considered successes, under the assumption that they maintained good tolerance at home. In the PP population, from which subjects transferred to another hospital or discharged before the 21st day are excluded, the occurrence of death, necrotizing enterocolitis, and at least 1 episode of feeding intolerance were regarded as failure.

The difference in the outcome between the 2 study arms was tested with the Fisher exact test. The risk of recurrent episodes of feeding intolerance (interruption of enteral feeding for at least 8 hours) in the 2 arms was estimated on the ARS population with the Andersen and Gill Cox's model for recurrent processes (23).

The analysis of secondary endpoints, because time-dependent, was carried out on PP population, resorting to generalized linear models (24): the number of episodes of feeding intolerance, feeding interruptions (any duration), bilious gastric residuals, and vomiting occurred during the observation were modeled as a Poisson variable; total hours of enteral feeding interruption were modeled, after log-transformation, as normal variables. Median time required to reach full enteral feeding was estimated on the ARS population according to Kaplan and Meyer (25). Body weight was expressed as SDS, with respect to Italian Neonatal Study (INeS) charts (26). To evaluate differences in growth between the 2 arms, weight gain was expressed as weight-ΔSDS/days, that is, the mean daily weight-SDS variation between SDS on the first day of exclusive enteral feeding and discharge. In this analysis, only babies discharged home were considered.

SAS software was used to process data and fit statistical models (18).

## RESULTS

**FIGURE 1:**
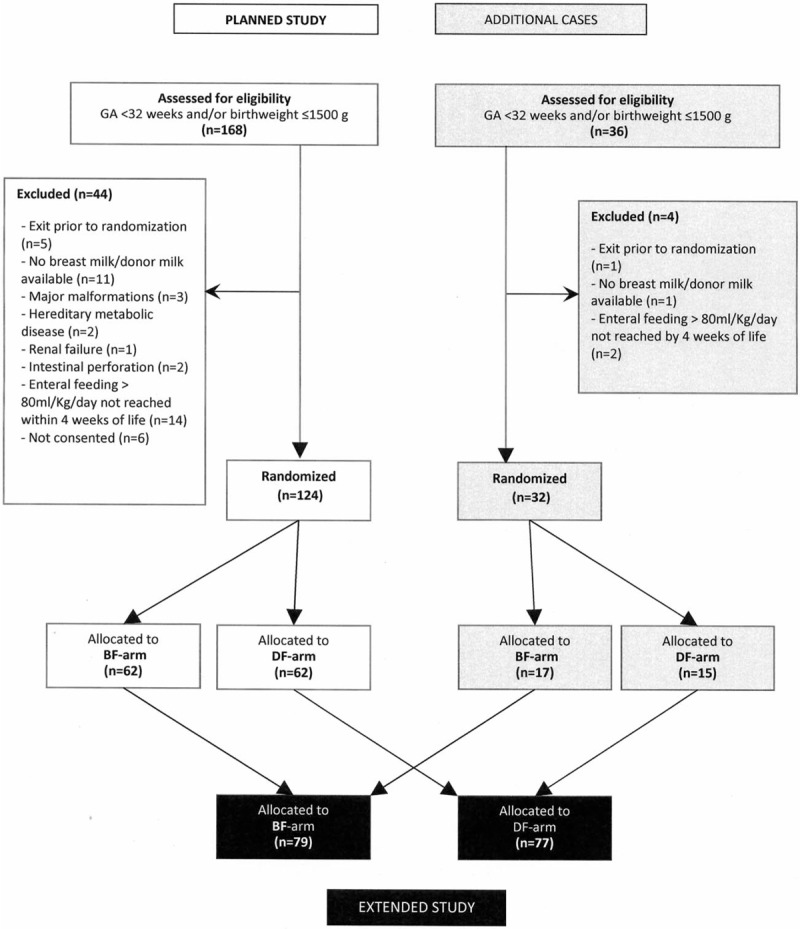
Diagram of the enrollment, randomization, and study allocation. BF = bovine milk–derived fortifier; DF = donkey milk–derived fortifier.

The ARS population consisted of 124 newborns enrolled in the planned study (BF-arm: n = 62; DF-arm: n = 62). During the extended study 32 more newborns were enrolled, for a total of 156 babies (BF-arm: n = 79, DF-arm: n = 77) (Fig. 1). The PP population (patients that completed 21 days of observation) was made up of 89 newborns (BF-arm: 44, DF-arm: 45) in the planned study and 111 (BF-arm: 57, DF-arm: 54) in the extended study. No babies switched from one arm to the other.

Table 1 shows the characteristics of mothers and neonates included in the planned and extended study before enrollment and clinical outcome and morbidities that occurred during the observation period. In the table, PDA refers to a condition from which the newborn recovered before randomization. The median time lag between the random assignment of subjects to either arms and the actual start of the intervention did not exceed 3 days. One baby per arm died following necrotizing enterocolitis.

### Primary Endpoint

**FIGURE 2:**
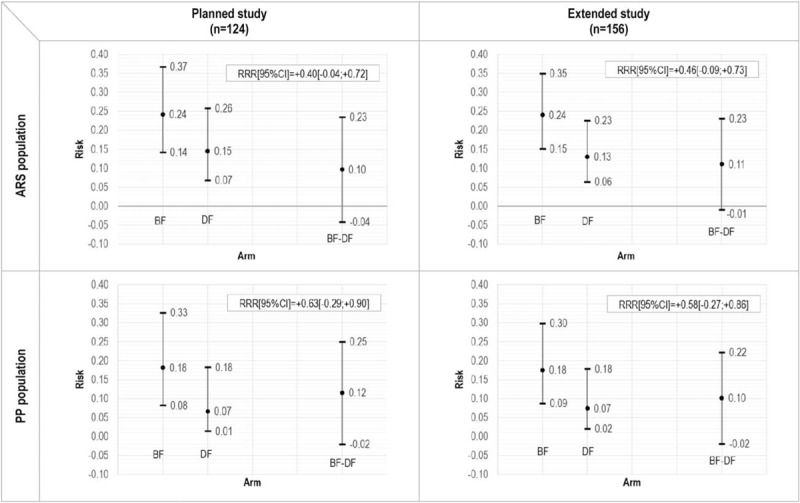
Primary endpoint: risk of failure in the 2 arms and relative risk reduction (RRR), and 95% confidence intervals. ARS = all randomized subjects; PP = per-protocol.

The number of failures and successes observed in the 2 arms for the planned and the extended study is reported in Supplementary Table 1 (top) (Supplemental Digital Content). Risk of failure in the planned study tended to be lower in the DF- than in the BF-arm, with a relative risk reduction of 0.40 (95% confidence interval [CI]: −0.27, +0.72; Fisher exact test: *P* = 0.256) in the ARS and 0.63 (95% CI: −0.29, +0.90; *P* = 0.118) in the PP population (Fig. 2, left). Results were similar in the extended study, with relative risk reductions of 0.46 (95% CI: −0.09, +0.73; *P* = 0.100) in the ARS and 0.58 (95% CI: −0.27, +0.86; *P* = 0.153) in the PP population (Fig. 2, right).

### Secondary Endpoints

**FIGURE 3:**
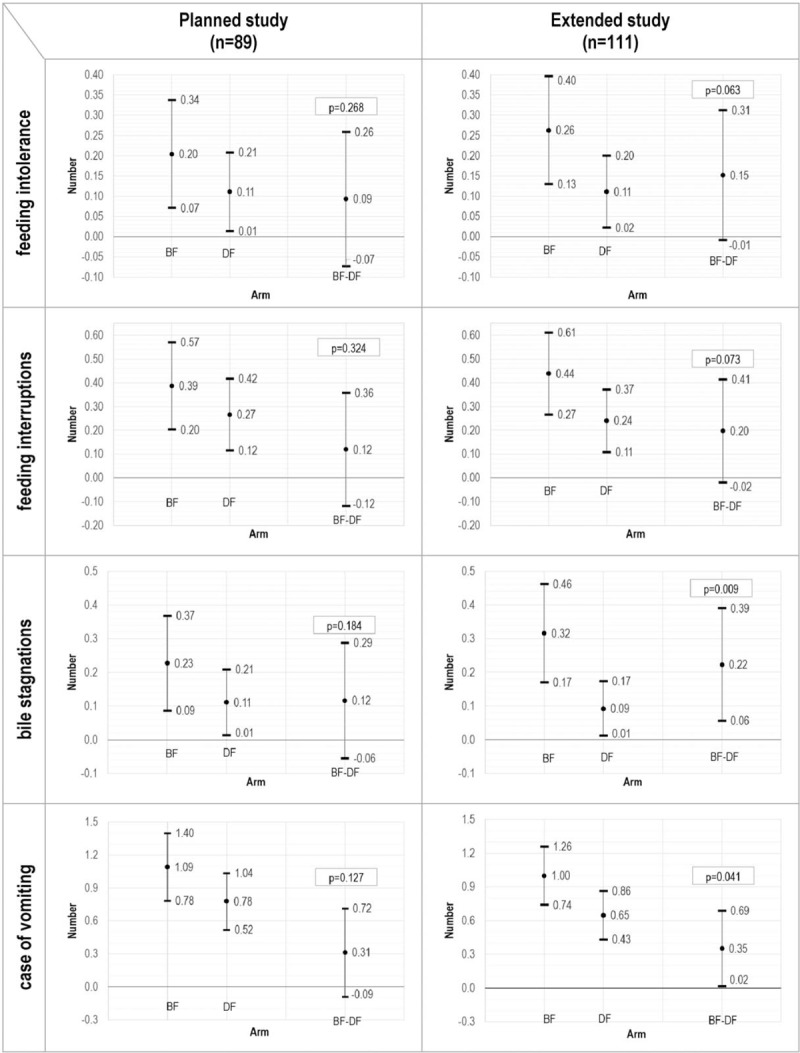
Secondary endpoints (on PP population): means, mean differences, and 95% confidence intervals.

The number of episodes of feeding intolerance, feeding interruptions (any duration), bilious gastric residuals, and vomiting for the planned and extended study observed in the PP population is reported in Supplementary Table 1 (bottom) (Supplemental Digital Content). During the observation period, the mean number of episodes per newborn of these secondary endpoints was consistently lower in the DF- than in the BF-arm. Indeed, the difference between the BF- and the DF-arm ranged from 0.09 to 0.31 in the planned study (Fig. 3, left), and from 0.15 to 0.35 in the extended study (Fig. 3, right). In the extended study, the difference between the arms was statistically significant as regards the number of episodes of bilious gastric residuals (*P* = 0.009) and vomiting (*P* = 0.041).

The hazard ratio of recurrent feeding intolerance episodes (DF-arm vs BF-arm), estimated in the ARS population using Anderson and Gill Cox's model for recurrent processes, was 0.53 (95% CI: 0.20, 1.44; *P* = 0.215) in the planned study, and 0.40 (95% CI: 0.17, 0.95; *P* = 0.038) in the extended study.

The median time to achieve full enteral feeding in the BF- and DF-arms was 19 days (95% CI: 15, 23), both in the planned and in the extended study. The total number of hours of feeding interruptions did not differ significantly between the 2 arms, neither in the planned study (BF-arm: 1.28; 95% CI: 0.50, 2.49 and DF-arm: 0.68; 95% CI: 0.10, 1.55; *P* = 0.304) nor in the extended study (BF-arm: 1.15; 95% CI: 0.49, 2.12 and DF-arm: 0.66; 95% CI: 0.14, 1.44; *P* = 0.340).

Mean daily weight increase between the first day of exclusive enteral feeding and discharge (expressed as ΔSDS/day) did not differ between the BF-arm (−0.013; 95% CI: −0.018, −0.009) and the DF-arm (−0.012; 95% CI: −0.016, −0.008) in the planned study. Similar results were observed in the extended study (BF-arm: −0.012; 95% CI: −0.016, −0.008; DF-arm: −0.013; 95% CI: −0.018, −0.008).

## DISCUSSION

The aim of the study was to assess the effects of a donkey milk–derived human milk fortifier on feeding tolerance among very preterm (gestational age <32 weeks) and VLBW (≤1500 g) newborns. To the best of our knowledge, our trial is the first to investigate the use of a DF for the nutrition of very preterm and VLBW newborns. All newborns (both the BF-arm and the DF-arm) received human milk exclusively (raw own mother's milk or pasteurized donor milk), without any preterm bovine formula supplementation. In contrast, Sullivan et al (27) included subjects receiving preterm formula in the group supplemented with the bovine fortifier in their comparison of a human milk–based and bovine milk–based fortifier, which represents a confounding variable.

In our study, we observed a lower number of failures (necrotizing enterocolitis, at least 1 episode of feeding intolerance, or death) and a lower hazard of feeding intolerance episodes in the DF-arm, both in the planned and in the extended study, in ARS and in PP population. The mean number of episodes per newborn of feeding intolerance, feeding interruptions (any duration), bilious gastric residuals, and vomiting during the observation period was consistently lower in DF-arm, both in the planned study and in the extended study. Overall, these results suggest the favorable effect of the donkey milk fortifier on feeding tolerance, which could not be demonstrated due to the unexpected lack of power of our study. Actually, our study was planned under the assumption that the occurrence of failures (necrotizing enterocolitis, at least 1 episode of feeding intolerance, or death) in the control arm (BF-arm) was 45%, whereas during the trial it was only 24%. This could be due to the so-called *Hawthorne effect*(28,29), that is, to the fact that the behavior of clinical staff may be affected and improved when a trial is conducted in a clinical setting. Because of the lower occurrence of failures, the statistical power to detect a decrease from 24% to 11% (ie, the same relative decrease in failure occurrence assumed in the protocol), was only 38% (about half of the prefixed 80%) in the planned study and 48% in the extended study. Under these conditions, it would have been necessary to enroll 148 subjects per arm to achieve an 80% power.

Overall, a better tolerance of DFs emerged. We speculate that the quality of donkey milk protein could be responsible of this result, the 2 diets being isoproteic and isocaloric. Weight gain was similar in BF- and DF-arms, suggesting that differences in tolerance do not affect short-term growth, at least under the conditions on which this trial was carried out, where a parenteral intake was provided in case of episodes of enteral feeding intolerance and suspension. For this reason, a similar total nutritional intake was provided in all subjects. At present, commercially available fortifiers are bovine milk derived, with a protein composition that is very different from that of human milk. Bovine milk whey proteins contained in the fortifier used in this study strongly differ from human milk counterparts in term of relative abundance and primary structure (30). The intake of bovine milk protein in the first months of life has raised concerns because of its association with allergies (31). Furthermore, bovine milk has been reported as a possible trigger of intestinal inflammation in preterm neonates (32,33). Previously, we found that the protein and lipid fractions in donkey milk are similar to those in human milk (30,34). We also observed that donkey milk was well tolerated in a group of children with highly problematic cow's milk allergies (35). Moreover, it has recently been demonstrated in murine models that a supplementation of the basal diet with donkey milk decreases the accumulation of body lipids and affects glucose and lipid metabolism in a manner more similar to human than to bovine milk. These biological effects are comparable to those elicited by human milk (16,17). Based on the above-mentioned studies and the results obtained in the present trial, it can be hypothesized that donkey milk is more suitable than bovine milk as an ingredient in human milk fortifier for very preterm and VLBW newborns.

For a more comprehensive evaluation of the results, we should consider that the 2 arms slightly differed: a higher number of newborns developed PDA in the BF-arm before randomization (and PDA at the time of randomization was an exclusion criterion), small for gestational age (SGA) newborns were more frequent in the DF-arm, whereas VLBW newborns were more frequent in the BF-arm.

The presence of symptomatic PDA may theoretically impair feeding tolerance because of the impact on blood flow to vital organs (36,37), but at time of randomization this condition had been resolved. SGA newborns are at higher risk for intestinal disturbances, ranging from temporary enteral feeding intolerance to necrotizing enterocolitis. In our study, the best tolerance was observed in the DF-arm, in which SGA subjects, who were at major risk of feeding difficulties, were more numerous.

A limitation of this trial is that it was designed as an open-label randomized clinical trial, because the nurses in charge of the preparation of meals were also in charge of evaluating signs of feeding tolerance. The nurses, however, involved in the trial should stick to a strict protocol to reduce their discretion in the evaluation of signs of feeding intolerance.

To conclude, the new DF was well tolerated in our population. The results of this trial may constitute a sound basis on which to plan a further trial with enough power to confirm the higher tolerability of the DF and open new perspectives for the production of human milk fortifiers other than those derived from bovine milk.

## Supplementary Material

Supplemental Digital Content

## Figures and Tables

**TABLE 1:** Maternal and neonatal characteristics, clinical condition at randomization, and clinical outcome and morbidities during the observation period

		Planned study		Extended study	
		BF-arm (n = 62)	DF-arm (n = 62)	BF-arm (n = 79)	DF-arm (n = 77)
Maternal characteristics					
Pregravidic BMI, kg/m^2^	Mean (SD)	23.7 (4.53)	23.6 (5.70)	23.4 (4.47)	24.0 (5.96)
Weight gain in pregnancy, kg	Mean (SD)	9.0 (6.02)	8.2 (6.14)	8.7 (6.00)	8.8 (5.94)
Age, y	Median (IQR)	33.5 (30–38)	34.5 (30–39)	34 (30–38)	34 (30–39)
Chronic diabetes	n (%)	0 (0.0)	1 (1.6)	0 (0.0)	1 (1.3)
Chronic hypertension	n (%)	3 (4.8)	2 (3.2)	3 (3.8)	3 (3.9)
Gestational diabetes	n (%)	10 (16.1)	11 (17.7)	11 (13.9)	14 (18.2)
Gestational hypertension	n (%)	18 (29.0)	11 (18.0)	22 (27.8)	12 (15.8)
Caesarean delivery	n (%)	50 (80.6)	46 (74.2)	58 (73.4)	60 (77.9)
Prelabor rupture of membranes	n (%)	17 (27.4)	15 (24.2)	23 (29.1)	17 (22.1)
Assisted reproductive technology	n (%)	15 (24.2)	12 (19.4)	19 (24.1)	13 (16.9)
Neonatal characteristics					
Boys	n (%)	29 (46.8)	31 (50.0)	36 (45.6)	37 (48.1)
Singletons	n (%)	35 (56.5)	40 (64.5)	47 (59.5)	46 (59.7)
Firstborn	n (%)	40 (64.5)	39 (62.9)	51 (64.6)	50 (64.9)
Gestational age <32 wk^*^	n (%)	50 (80.6)	48 (77.4)	64 (81.0)	55 (71.4)
VLBW (birth weight ≤1500 g)^†^	n (%)	57 (91.9)	53 (85.5)	70 (88.6)	65 (84.4)
Small for gestational age^‡^	n (%)	16 (25.8)	20 (32.3)	19 (24.4)	27 (35.1)
Weight, g	Mean (SD)	1166 (297.3)	1196 (315.7)	1161 (310.3)	1214 (311.5)
Weight (SDS)	Mean (SD)	−0.36 (1.122)	−0.64 (1.165)	−0.35 (1.120)	−0.74 (1.162)
RDS	n (%)	53 (85.5)	54 (87.1)	69 (87.3)	67 (87.0)
Age at randomization, days	Median (IQR)	9.0 (6–17)	8.5 (5–14)	9.0 (6–17)	8.0 (5–14)
Age at start of intervention, days	Median (IQR)	11.5 (8–17)	10.5 (7–17)	12.0 (8–18)	11.0 (7–17)
Intraventricular hemorrhage	n (%)	5 (8.1)	2 (3.2)	8 (10.1)	3 (3.9)
Recovered patent ductus arteriosus	n (%)	20 (29.4)	11 (16.2)	26 (38.5)	11 (16.2)
Clinical outcome and morbidities					
Length of hospital stay^§^	Median (IQR)	45 (32–63)	39.5 (29.5–63)	45.5 (32–63)	38 (28–56)
Transferred to other hospital	n (%)	6 (9.7)	5 (8.1)	7 (8.9)	5 (6.5)
Dead before discharge	n (%)	1 (1.6)	1 (1.6)	1 (1.3)	1 (1.3)
Steroids therapy	n (%)	1 (1.6)	1 (1.6)	1 (1.3)	1 (1.3)
Early sepsis	n (%)	3 (4.8)	1 (1.6)	4 (5.1)	1 (1.3)
Late sepsis	n (%)	4 (6.5)	3 (4.8)	5 (6.3)	3 (3.9)
Necrotizing enterocolitis	n (%)	1 (1.6)	1 (1.6)	1 (1.3)	1 (1.3)

BF = bovine milk–derived fortifier; BMI = body mass index; DF = donkey milk–derived fortifier; IQR = interquartile range; RDS = respiratory distress syndrome; SD = standard deviation; SDS = standard deviation score; VLBW = very-low-birth weight.

^*^Regardless of birth weight.

^†^Regardless of gestational age at birth.

^‡^Birth weight below the 10th centile of Italian Neonatal Study (INeS) charts [26].

^§^Computed on babies discharged to home.
