# Supplementary material for: A Novel Donkey Milk–derived Human Milk Fortifier in Feeding Preterm Infants: A Randomized Controlled Trial
Source: J Pediatr Gastroenterol Nutr. 2018 Oct 24;68(1):116–23. doi: 10.1097/MPG.0000000000002168 (PMC6314716; doi:10.1097/MPG.0000000000002168)
Supplement: Supplemental Digital Content [file jpga-68-116-s001.doc]

**Supplementary Table 1**

**Primary endpoint: number of success and failures by arm***

| **population** |  | **Planned study**  **(n=124)** | | **Extended study**  **(n =156)** | |
| --- | --- | --- | --- | --- | --- |
|  |  | **FAILURE** | **SUCCESS** | **FAILURE** | **SUCCESS** |
| **ARS** | **BF-arm** | 15 | 47 | 19 | 60 |
|  | **DF-arm** | 9 | 53 | 10 | 67 |
|  | ***TOTAL*** | *24* | *100* | *29* | *127* |
| **PP** | **BF-arm** | 8 | 36 | 10 | 47 |
|  | **DF-arm** | 3 | 42 | 4 | 50 |
|  | ***TOTAL*** | *11* | *78* | *14* | *97* |

BF= bovine fortifier; DF= donkey fortifier

* Failure is defined as occurrence of necrotizing enterocolitis, at least 1 episode of feeding intolerance (interruption of enteral feeding ≥ 8 consecutive hours), or death. In the ARS population failure also included transfer to another hospital before the 21^th^ day of observation.

**Occurrence of secondary endpoints.**

|  |  | **Planned study**** | | **Extended study**** | |
| --- | --- | --- | --- | --- | --- |
|  | **n of episodes** | **n of subjects** | | **n of subjects** | |
|  |  | **BF-arm**  **(n=44)** | **DF- arm**  **n=45)** | **BF- arm**  **(n=57)** | **DF- arm**  **(n=54)** |
| **feeding intolerance** | **1** | 7 | 1 | 8 | 2 |
|  | **2** | 1 | 2 | 1 | 2 |
|  | **5** | 0 | 0 | 1 | 0 |
| **feeding interruptions** | **1** | 6 | 6 | 7 | 7 |
|  | **2** | 2 | 3 | 2 | 3 |
|  | **3** | 1 | 0 | 1 | 0 |
|  | **4** | 1 | 0 | 1 | 0 |
|  | **7** | 0 | 0 | 1 | 0 |
| **bilious gastric residuals** | **1** | 3 | 1 | 3 | 1 |
|  | **2** | 1 | 0 | 1 | 0 |
|  | **4** | 0 | 1 | 0 | 1 |
|  | **5** | 1 | 0 | 1 | 0 |
|  | **8** | 0 | 0 | 1 | 0 |
| **vomiting** | **1** | 8 | 8 | 11 | 8 |
|  | **2** | 7 | 5 | 8 | 5 |
|  | **3** | 3 | 1 | 3 | 1 |
|  | **4** | 0 | 1 | 1 | 1 |
|  | **5** | 2 | 2 | 2 | 2 |
|  | **7** | 1 | 0 | 1 | 0 |

BF= bovine fortifier; DF= donkey fortifier

** Observed in PP population.

**Supplementary Table 2**

**Planned study: number of patients in the BF-arm, by gestational age and birthweight**

|  | **Gestational Age** | | |
| --- | --- | --- | --- |
| **Birthweight** | **<32 w** | **≥32w** | **Total** |
| **≤1500** | 45 | 12 | 57 |
| **>1500** | 5 | 0 | 5 |
| **Total** | 50 | 12 | 62 |

**Planned study: number of patients in the DF-arm, by gestational age and birthweight**

|  | **Gestational Age** | | |
| --- | --- | --- | --- |
| **Birthweight** | **<32 w** | **≥32w** | **Total** |
| **≤1500** | 39 | 14 | 53 |
| **>1500** | 9 | 0 | 9 |
| **Total** | 48 | 14 | 62 |

**Extended study: number of patients in the BF-arm, by gestational age and birthweight**

|  | **Gestational Age** | | |
| --- | --- | --- | --- |
| **Birthweight** | **<32 w** | **≥32w** | **Total** |
| **<=1500** | 55 | 15 | 70 |
| **>1500** | 9 | 0 | 9 |
| **Total** | 64 | 15 | 79 |

**Extended study: number of patients in the DF-arm, by gestational age and birthweight**

|  | **Gestational Age** | | |
| --- | --- | --- | --- |
| **Birthweight** | **<32 w** | **≥32w** | **Total** |
| **≤1500** | 43 | 22 | 65 |
| **>1500** | 12 | 0 | 12 |
| **Total** | 55 | 22 | 77 |

BF= bovine fortifier; DF= donkey fortifier
